# The rise in child injuries involving all-terrain vehicles in Israel: a call to action

**DOI:** 10.1186/s13584-025-00739-8

**Published:** 2025-12-10

**Authors:** Aviad Agam, Francis B. Mimouni, Yigal Godler, Elad Calif, Joseph Mendlovic

**Affiliations:** 1 Beterem Safe Kids Israel, Halapid 10, Petach Tikvah, Israel; 2 Faculty of Medicine, Tel Aviv University, Tel Aviv, Israel; 3 The Research Institute, Leumit Health Services, Tel Aviv, Israel; 4 Shaare Zedek Medical Center, Affiliated with the Hadassah-Hebrew University School of Medicine, Jerusalem, Israel; 5 Israel Ministry of Health, Jerusalem, Israel

**Keywords:** All-Terrain vehicles (ATVs), Child injuries, Regulatory policy, Safety technologies, Israel

## Abstract

**Background:**

The first five months of 2025 marked a sharp and troubling increase in child injuries involving All-Terrain Vehicles (ATVs) in Israel. During this short period, 17 children were reported as injured or killed in ATV-related incidents – three fatally - with a mean age of 12.5 years (median: 14). This represents a 263% rise compared to previous annual rates, highlighting serious concerns regarding the effectiveness of existing legislation, enforcement and public awareness. The purpose of this study is to analyze long-term trends in ATV-related child injuries and assess the urgent need for regulatory and preventive interventions.

**Methods:**

We conducted a retrospective analysis of unintentional childhood injuries involving ATVs using Beterem Safe Kids Israel’s media-based pediatric injury database, from 2008 until May 2025. Trends over time were analyzed using best-fit regression models, and group differences were tested using chi-square tests of independence.

**Results:**

Between 2008 and May 2025, 378 ATV-related injuries among children and adolescents were documented, including 41 fatalities. A clear upward trend was observed, with the annual average rising from 21.9 cases per year (2008–2019) to 43 cases per year (2020–2025), and with 22 cases already reported in 2025. Severe and fatal cases more than doubled during this period. Arab children were overrepresented relative to their population share. Incidents peaked during weekends and holidays. A significant quadratic increase in mortality rates over time was identified (R² = 0.751, *P* < 0.001). ATV-related injuries were disproportionately concentrated among children from lower socioeconomic backgrounds, with 95% of Arab victims and 74% of Jewish victims residing in low- or mid-ranking municipalities.

**Conclusions:**

ATVs present an escalating and preventable danger to child safety in Israel, with recent data signaling a critical and worsening trend. Immediate and decisive action is imperative. Policymakers must urgently implement a uniform minimum age for ATV operation, mandate safety technologies (e.g., Operator Protective Devices), require physical capability assessments, and launch targeted public awareness and enforcement campaigns—especially in rural and underserved communities. These measures are essential to safeguarding children and reversing the alarming trajectory of ATV-related harm.

## Introduction

The use of recreational all-terrain vehicles (ATVs) in Israel has steadily increased in recent years, particularly for sports and leisure activities [1]. It is estimated that approximately 680 ATVs or Quad bikes are sold annually in Israel, with 12% used for sports, 32% for beginner training, and 56% for leisure purposes [2]. This growing popularity has raised significant concerns regarding the risks these vehicles pose - especially to children and adolescents. These concerns are particularly acute in rural areas, where ATVs are frequently used not only for recreation but also for agricultural and occupational purposes [3].

In the US, according to the U.S Consumer Product Safety Commission (SPSC), off highway vehicles cause 800–900 fatalities per year, with ATV alone accounting for about two-third of these fatalities [4]. From 2000 to 2007, average fatality rate was 0.32 deaths per 100,000 people, with men six times more likely to die than women, with the highest risk among young adults aged 15–44 [5]. It is estimated that over 100,000 people visit USA emergency rooms yearly due to ATV-related injuries [6].

In Israel, despite the growth in ATV use, national regulation and enforcement remain limited compared with other high-income countries (see Discussion section). Safety requirements such as the installation of Operator Protective Devices (OPDs) were recently revoked, and significant regulatory gaps persist regarding electric ATVs marketed for children. These gaps highlight the absence of a comprehensive policy framework to address ATV-related risks in Israel.

This paper responds to a marked increase in child injuries involving ATVs reported in Israel during the first five months of 2025. This emerging trend underscores the urgent need for state authorities to intervene and reverse the pattern by promoting safer practices in ATV use. To this end, we present an analysis of long-term trends based on Beterem Safe Kids Israel’s media-based injury database and propose several action items aimed at preventing injuries among children and adolescents.

## Methods

Data on unintentional childhood injuries involving all-terrain vehicles (ATVs) were obtained from the media-based pediatric injury database (individuals under 18 years of age), maintained by Beterem Safe Kids Israel since 2008. This database is updated weekly and complies detailed reports of external-cause childhood mortality and morbidity in Israeli print and online media, in Hebrew, Arabic, English and Russian [7–9]. Cases are classified according to the World Health Organization’s Minimum Data Set (MDS) for injury and mortality documentation [10].

For this study, all ATV-related injury and mortality cases involving children reported between January 2008 and May 2025 were extracted. Each case was individually reviewed to reconstruct the circumstances of the injury as fully as possible. When classifying injury severity, information was taken from the media report, which typically includes details on the degree of injury. In cases where severity was unclear or the child subsequently died, follow-up media reports and direct communications with the relevant hospital were used to confirm or supplement the data. This approach ensured that both non-fatal and fatal cases were classified as accurately as possible.

For each event, the following variables were documented: date, day of the week, time of day, location, victim’s age and gender, place of residence, ethnic group (Jewish/Arab), and socioeconomic ranking at the municipal level (based on Central Bureau of statistics (CBS) data, grouped into three clusters) [11]. Sectors classification was inferred from the victim’s residence and incident context. To prevent duplication, all cases are manually cross-refernced across multiple media sources, and any reports describing the same incident are identified and consolidate into a single entry. Data were processed using Microsoft Excel, resulting in a total of 378 injury cases, including 41 fatalities. Finally, the sample size of child population in Israel at any given year was extracted from the annual CBS publications [12].

To evaluate the statistical significance of the presented results, we conducted a chi-square test of independence. A P-value of < 0.05 was deemed significant.

We used the best-fit regression function of the Minitab Statistical Package (version 17, State College, PA) to examine trends in the frequency of ATV-related injuries over the study period. Several model specifications were evaluated, including linear and higher-order polynomial regressions. Based on visual inspection of the data and model diagnostics, a quadratic regression model was selected as the best fit. This model captured the non-linear pattern observed in the data, an initial rise followed by a decline in injury rates, while providing a superior fit (as indicated by the adjusted R-squared and residual analysis) compared to the linear model, and avoiding the overfitting risk associated with higher-order terms. This approach allowed for a parsimonious yet accurate representation of temporal changes in injury frequency.

## Results

**Fig. 1:**
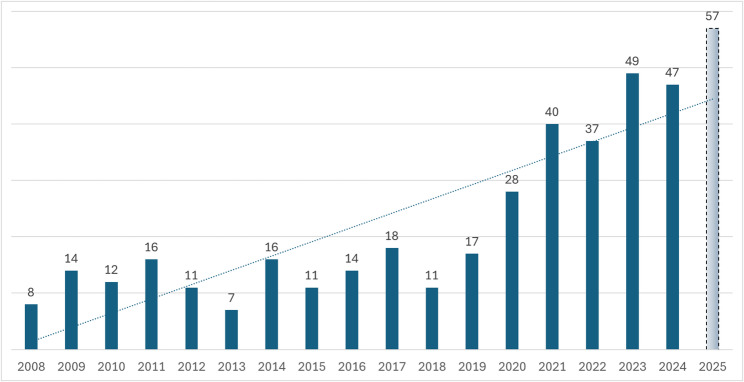
Child injury involving ATVs over time. The 2025 figure is an estimate based on the current rate, and should be treated with caution

Between 2008 and May 2025, a total of 378 cases of children and adolescents injured by ATVs were documented. Over the years, there was a clear upward trend in such injuries (Fig. 1). From an average of 12.9 reported cases per year between 2008 and 2019, the average rose to 43 reported cases per year in the past six years (2020–2025) – an increase of 233%. As of May 22, 2025, 22 cases have already been reported for the year. Based on this rate, the projected annual total is 57 cases. As the rate of population growth is approximately 2% every year (CBS, 2023), the growth rate of ATV-related incidents clearly exceeds that of child population growth.

**Fig. 2:**
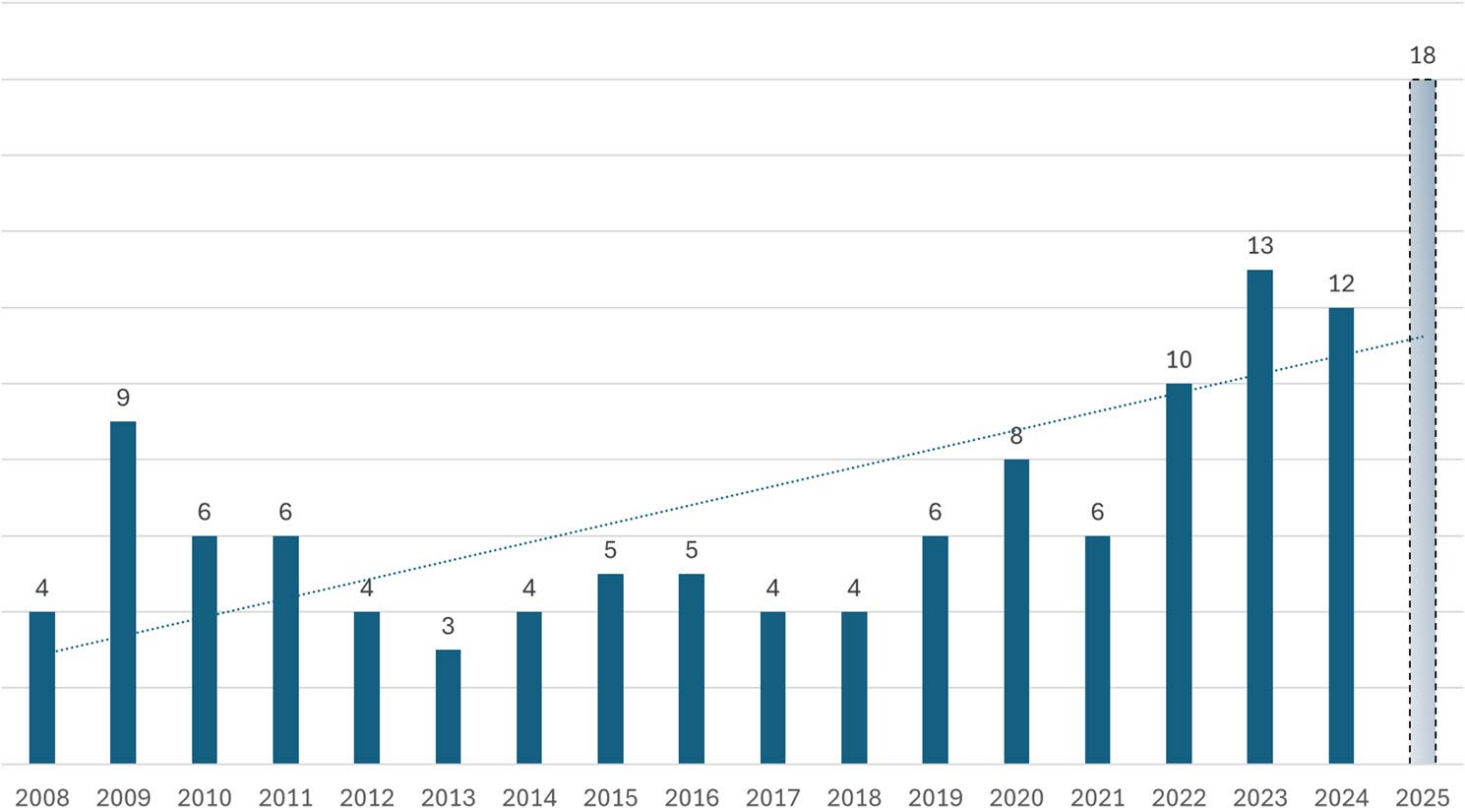
Annual distribution of severe injuries among children. The 2025 figure is an estimate based on current rate, and should be treated with caution

A similar trend is observed when focusing only on cases that resulted in severe or critical injury or death (Fig. 2), with a 123% increase in the annual average – from an average of 4.8 cases (2015–2019) to 11.2 cases per year (2020–2025). Table 1 breaks down injuries by severity, showing that the majority of cases were classified as moderate (*n* = 159). In total, 41 children died as a result of ATV-related incidents between 2008 and 2025, including three deaths reported in 2025.

**Table 1:** Annual distribution of child and adolescent injuries involving ATVs in Israel (2008–2025), by injury severity

Year	No Injuries	Minor	Minor/Moderate	Moderate	Moderate/Severe	Severe	Fatal	Unknown	Total
2008		1		3		4			8
2009		3		2		9			14
2010		2		3		4	2	1	12
2011		3		5		4	2	2	16
2012				4		2	2	3	11
2013				2		2	1	2	7
2014		2		6		3	1	2	16
2015		1		4	2	2	1	1	11
2016		2		5	1	2	2	2	14
2017		5	1	7		1	3	1	18
2018		2	2	2	1	1	2	1	11
2019		5	3	3	1	1	4		17
2020		4		13		6	2	3	28
2021		11	2	21	1	1	4		40
2022		11		15	1	6	3	1	37
2023		9		24		10	3	3	49
2024	2	7		26		6	6		47
2025		1		14		4	3		22
Total	2	69	8	159	7	68	41	6	378

**Fig. 3:**
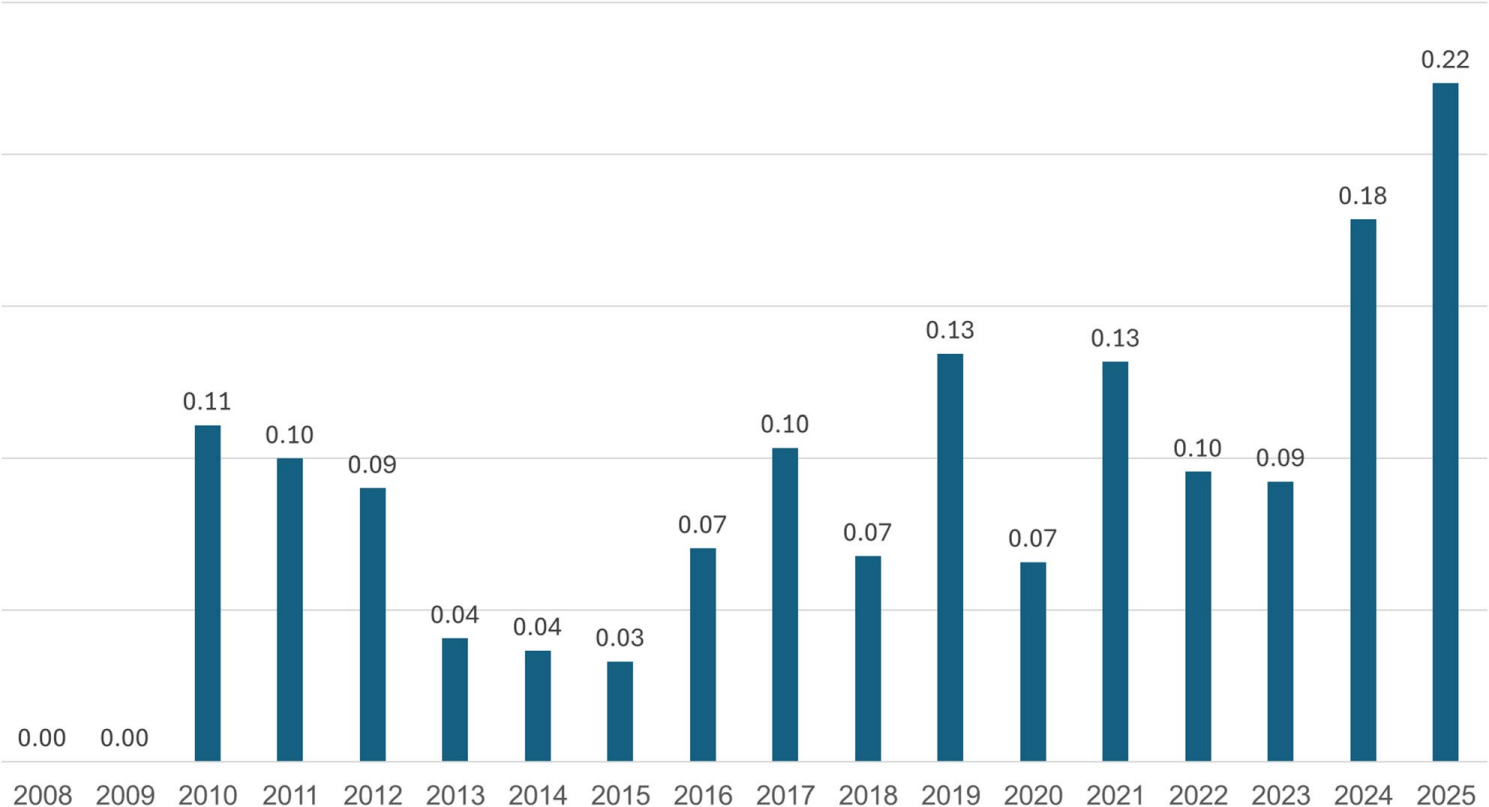
Mortality rates per year and per 100,000 children of ATV-related incidents over time

When looking only at fatalities and normalizing the data by child population size, a clear increase in mortality rate is evident in 2025 (Fig. 3). The current mortality rate in 2025 represents a 25% rise compared to 2024, and a 115% increase relative to the 2019–2023 average.

**Fig. 4:**
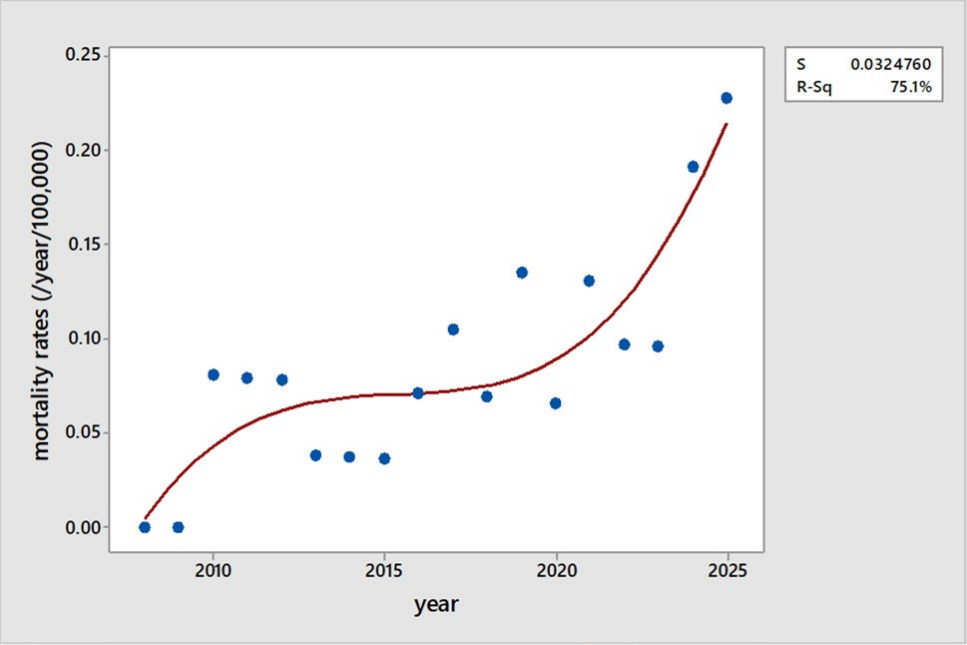
The trend in child mortality rate involving all-terrain vehicles (ATVs) over time. The figure illustrates a quadratic increase in ATV-related child mortality over the years, based on the best-fit regression model

Figure 4 shows that the child mortality rate involving ATV over the years increased in a quadratic fashion, according the best-fitted following regression equation: Mortality (number/year/100,000 children) = −1,252,096 + 1864 (year)−0.9248 year^2^ + 0.000153 year^2^ (R^2^ = 0.751, *P* < 0001).

**Fig. 5:**
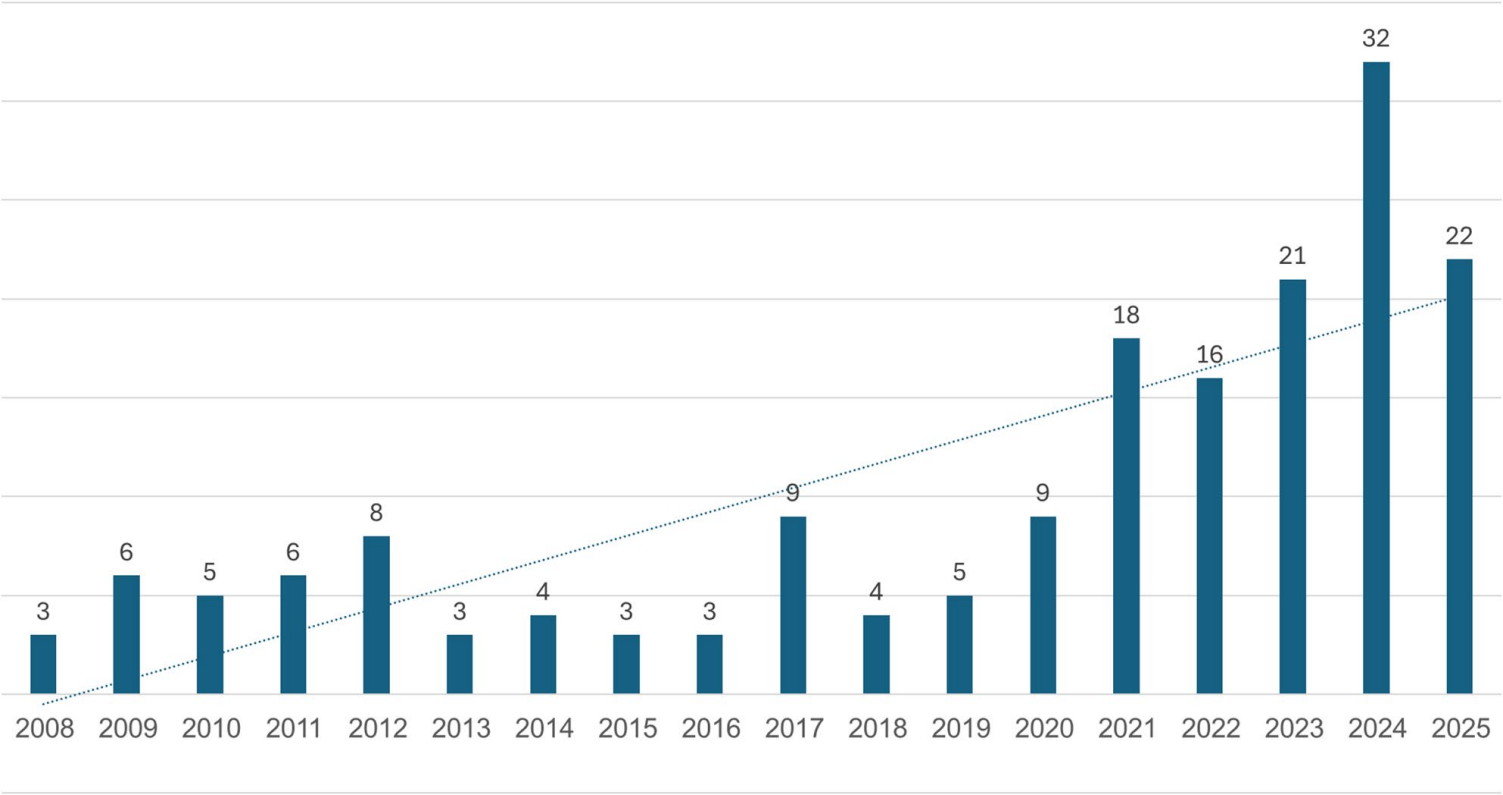
Cases of injury involving ATVs during the months January to May of each year

When comparing only the first five months of each year, 2025 shows the highest number of cases (*n* = 22), excluding 2024, which saw an unusual concentration of incidents early in the year (32 cases reported in the first five months) (Fig. 5).

**Fig. 6:**
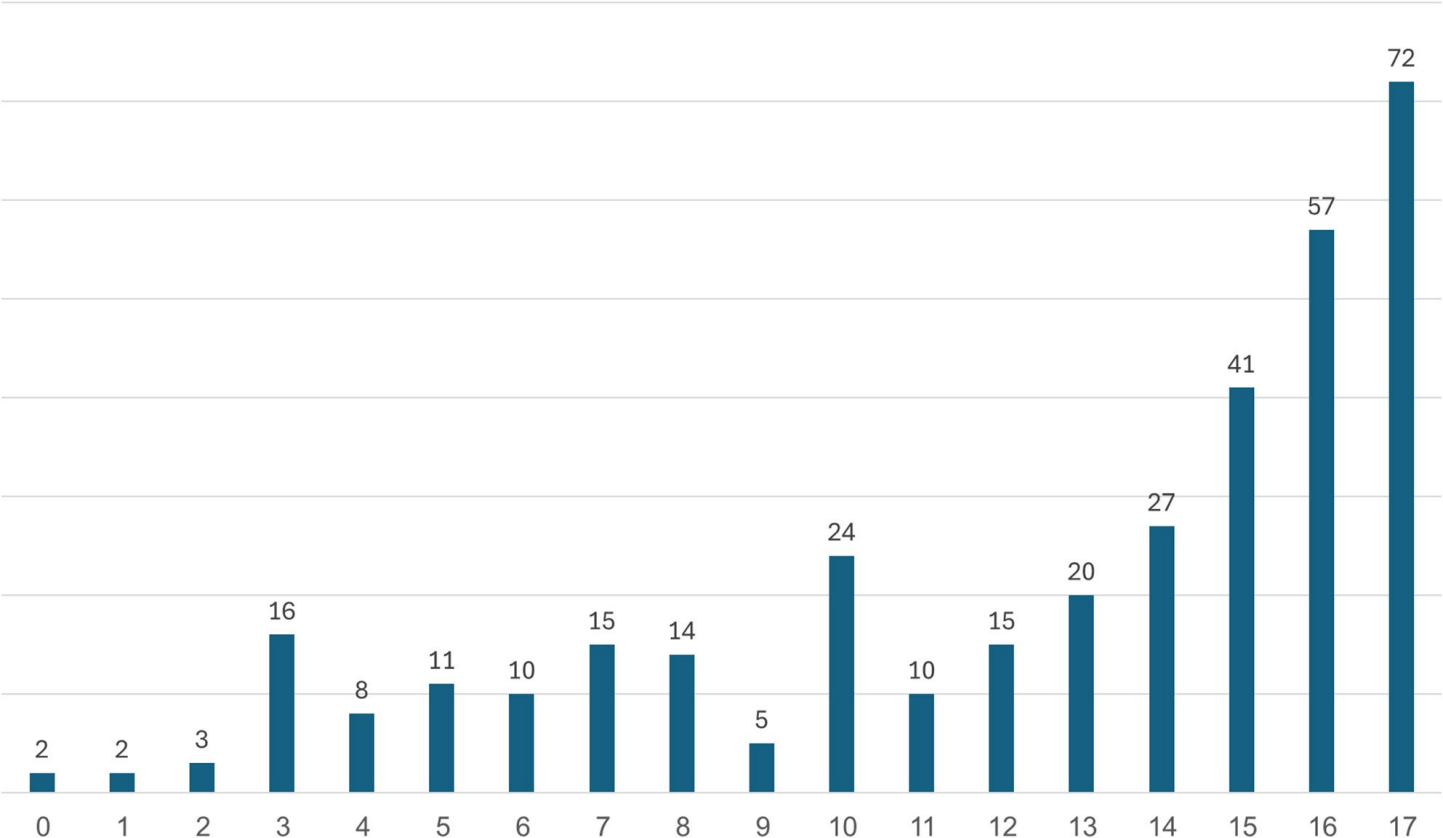
Distribution of cases of injury involving ATVs by the age of the victim

Of the 368 cases, 324 (86%) involved male victims. This aligns closely with injury patterns observed in incidents involving two-wheeled vehicles in general, and electric two-wheelers in particular, where males also constitute the vast majority of victims (Agam et al., under review). The average age of those injured is 12.5 years (median: 14), with 16- and 17-year-olds being the most commonly affected age groups (Fig. 6). In 26 cases, the victim’s age was not reported.

**Fig. 7:**
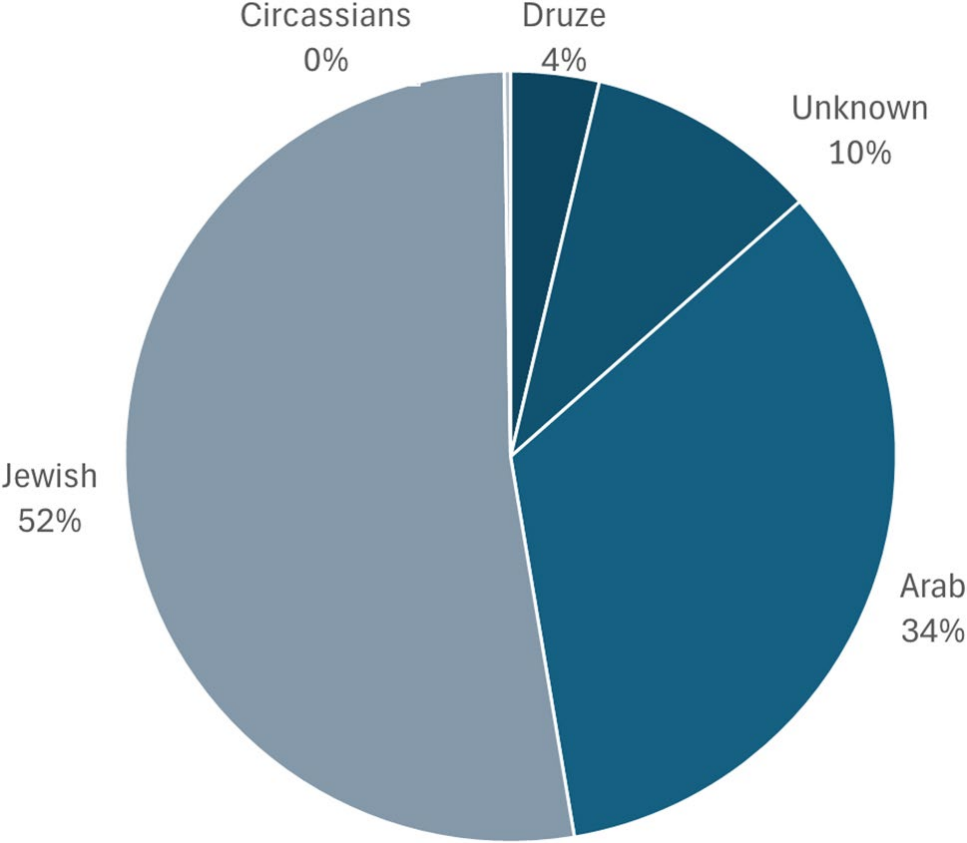
Distribution of cases of injury involving ATVs by ethnicity

Jewish children make up the majority of injured cases (*n* = 198; 52%) (Fig. 7). Children of Arab descent account for 34% of cases (*n* = 128), a proportion that exceeds their share of the child population in Israel.

Some differences as well as similarities emerge when comparing the Arab and Jewish populations. The average age of Jewish children injured in ATV-related incidents is 12.6 (median: 14), which is similar to that of Arab children (Average: 11.9; median: 14). The proportion of male victims is higher among Arab children (87.9%) compared to Jewish children (81.8%). Pedestrian victims are slightly more common in the Arab population (12%) than in the Jewish population (9%). Conversely, cases where the injured child or adolescent was the driver are slightly more frequent among Jewish children (29.8% vs. 27.3%).

**Fig. 8:**
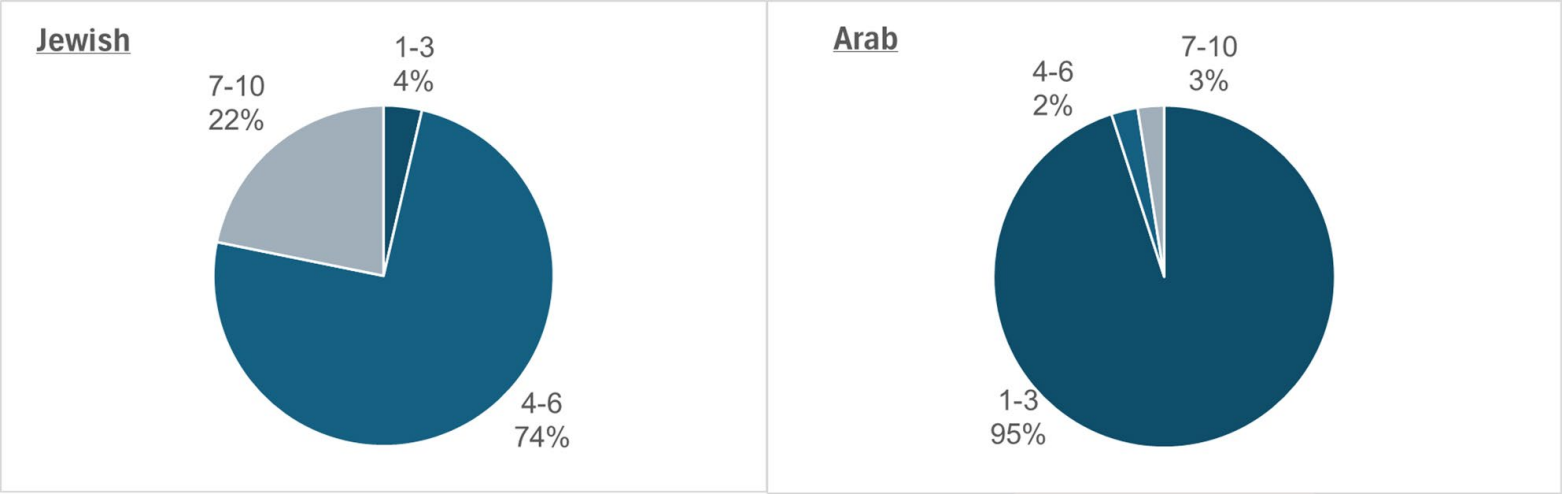
Distribution of ATV-related reported child injuries by sector and socioeconomic cluster

When examining the data by socioeconomic status, clear disparities are evident. Among Jewish cases, 74% of injured children reside in municipalities with a mid-level socioeconomic ranking, while 95% of Arab cases involve children from low-level socioeconomic municipalities (Fig. 8). However, this pattern should be interpreted with caution, as most Arab communities in Israel are classified as low-SES and are also predominantly rural. Therefore, the concentration of cases in these areas likely reflects a combination of factors, including socioeconomic disadvantages, rural geography that facilitates ATV use, and differing recreational norms and enforcement levels, rather than socioeconomic status alone. These overlapping contextual factors highlight the importance of considering both environmental and social determinants when addressing ATV-related injury risks among children.

**Fig. 9:**
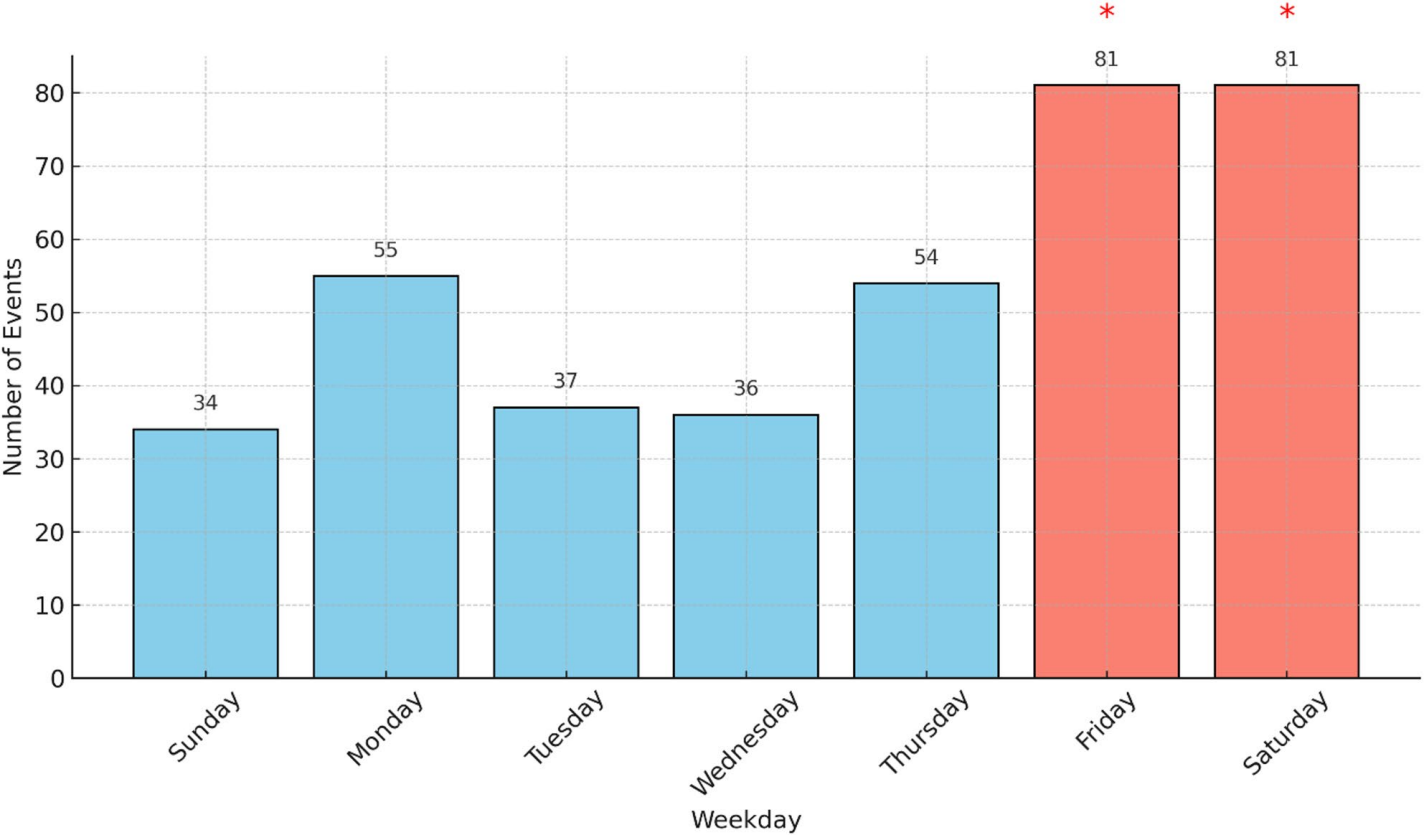
Distribution of ATV-related accidents by weekday. Bars represent the number of incidents reported per day. Friday and Saturday, marked with asterisks (), show statistically significant elevations compared to an even weekday distribution (χ²= 45.78, *p* < 0.001).*

Analysis of the distribution of ATV-related incidents across weekdays revealed a statistically significant deviation from a uniform distribution (χ² = 45.78, *p* < 0.001) (Fig. 9). While the expected number of events per day, assuming equal distribution, was 54, the observed frequencies were markedly higher on Fridays and Saturdays (81 events each, 21% per day) and lower on Sundays (34 events, 9%). This pattern suggests a concentration of incidents during the weekend, likely reflecting increased recreational ATV use during these days.

Analysis of ATV-related injuries by ethnic group and holiday period, excluding 2025 (due to partiality) revealed distinct patterns. Among the Arab population (116 total cases), a pronounced peak was observed during the Ramadan month, accounting for 19 injuries (16.4%), and during the Summer Vacation, with 20 injuries (17.2%). The Jewish population (188 total cases) experienced the highest number of incidents during the Summer Vacation as well, with 39 injuries (20.7%), followed by the Jewish holidays of Hanukkah (5 injuries, 2.7%), Passover (5 injuries, 2.7%), and Sukkot (6 injuries, 3.2%). These distributions were statistically significant (χ² = 89.34, *p* < 0.001), indicating a strong association between ethnic group and the holiday timing of ATV-related injuries.

It is important to note, however, that Ramadan and the Summer Vacation cover longer durations than most other Jewish holidays. When normalized by length of the holiday, the highest daily injury rates were observed during Purim (0.12 injuries/day, 2 cases total, 1.1% of Jewish cases) and Sukkot (0.10 injuries/day, 12 cases total, 3.2%), despite their shorter durations. In contrast, although Ramadan (0.09 injuries/day, 19 Arab cases, 16.4%) and Summer Vacation (0.06 injuries/day, 20 Arab cases, 17.2%; 39 Jewish cases, 20.7%) contributed high total numbers, their per-day rates were lower, suggesting their prominence is partly due to greater exposure time rather than increased daily risk.

Finally, among cases where the victim’s place of residence was known, 59% of Jewish victims lived in cities and 41% in rural areas. In contrast, 67% of Arab victims were from rural areas, while only 17% lived in cities.

## Discussion

All-terrain vehicles (ATVs) are widely used worldwide for both agricultural and recreational purposes. Despite their popularity, ATVs pose substantial safety risks – especially to children - due to their structural instability, high center of gravity, and the physical and cognitive immaturity of young users. These risks may be further influenced by weak or inconsistent regulatory framework, limited law enforcement, and widespread informal or illegal use.

The findings presented in this study reveal a troubling rise in ATV-related injuries and fatalities among children in Israel, with a particularly sharp increase in recent years. This trend highlights the need for targeted interventions and policy reforms. By examining the current regulatory environment in Israel and comparing it with international approaches, this discussion aims to identify key risk patterns and explore evidence-based strategies that can reduce injuries and save lives.

The results highlight not only the growing scale of the problem but also the complex interplay of socioeconomic, geographic, and demographic factors that shape children’s vulnerability to ATV-related harm. In particular, the concentration of incidents among Arab children likely reflects both socioeconomic disadvantages and the predominantly rural nature of many Arab communities, where open terrain and limited regulation increase exposure to ATV-related risks.

### The state of affairs in Israel

In Israel, ATV use is predominantly recreational and has grown markedly in recent years. Sales of ATV’s in Israel have grown and usage has dramatically change: around 680 ATVs are sold annually in Israel, with 56% purchased for leisure, 12% for sports, and only 32% for beginner training [1].

Operator Protective Devices (OPDs) are structural safety features designed to reduce the risk of severe or fatal injuries in rollover incidents by creating a protective zone around the rider, such as roll bars or cages. In Israel, the installation of OPD was previously required for ATV approval and licensing. However, in December 2024, The Israeli Ministry of Transport revoked this requirement, citing concerns regarding vehicle design and usability [13]. The policy change contrasts with international recommendations, which emphasize the importance of such protective structures in preventing fatalities, particularly among inexperienced and young riders [3].

Further compounding the risks is a major legislative gap regarding electric ATVs. Although current regulations classify electric ATVs as toys if their maximum speed does not exceed 16 km/h, in practice, much faster and more powerful models are routinely marketed and sold to children as young as four years old [14]. This regulatory loophole allows hazardous vehicles to circulate widely, without sufficient oversight, safety standards, or enforcement mechanisms – placing young riders and road users at considerable risk [3].

### Australia

Australia presents a contrasting example of proactive regulation. Since 2020, all new or imported ATVs must meet stringent safety standards, including the integration of OPDs, compliance with ANSI/SVIA 1–2017 or EN 15997:2011 standards [15], and mandatory rollover angle ratings displayed on consumer tags [16]. Empirical studies have shown that OPDs such as Quadbar and Lifeguard significantly reduce the risk of severe injury during rollover events [17]. Further research has underscored how minor terrain features—such as soil ruts or grass mounds—can destabilize ATVs, leading to loss of control and potentially fatal outcomes [18].

### Sweden

Sweden adopted a holistic strategy aimed at halving ATV-related fatalities and injuries by 2030 [19]. This includes mandatory helmet use, implementation of crush protection devices (CPDs), and deployment of technological innovations such as rollover warning systems. Public education campaigns and opinion surveys targeting forest workers and farmers are central to Sweden’s model [20]. Nevertheless, behavioral studies reveal that many parents believe their children can safely operate ATVs at a young age, often without supervision—highlighting a gap between safety recommendations and real-world practices [21].

### United States

In the United States, the American Academy of Pediatrics, surgical boards, and the Consumer Product Safety Commission (CPSC) broadly agree that children under six should neither operate nor ride as passengers on ATVs [4]. However, there is variation across states regarding guidelines for ages 6–15. While some jurisdictions permit the use of age-specific ATVs, others prohibit it altogether.

Recent research demonstrates that children - even when matched with appropriately sized vehicles—lack the physical strength, field of vision, and reach required for safe operation [21]. For example, most children cannot push an overturned ATV off their body—an essential self-rescue capability [22]. Among youth involved in agricultural education programs like FFA, the actual driving age was frequently below recommendations, with many beginning at ages 8–9. Alarmingly, nearly 35% of youth participating in this study reported involvement in at least one accident [23].

### Global ATV-related interventions

To inform policy in Israel, it is valuable to examine successful or promising approaches from other countries with comparable regulatory, demographic, or social contexts. This section highlights several community-based and educational interventions that have demonstrated measurable improvements in ATV safety knowledge, attitudes, and behavior among children and adolescents.

A study by House et al. [24], conducted in the United States, evaluated a brief video intervention demonstrating ATV performance and rollover scenarios featuring child “drivers”. Parents who viewed the video were significantly more likely to perceive ATVs as unsafe and to plan changes in household safety rules. This finding underscores the potential of short, evidence-based educational media tools to shift parental risk perception and promote safer practices around children.

In Iowa, USA, the Safety Tips for ATV Riders (STARs) classroom program was delivered in middle and high schools, teaching ten core ATV safety messages [25]. Evaluations involving over 4’600 students across 30 schools showed statistically significant short-term improvements in both knowledge and self-reported likelihood to follow safe riding behaviors. The program has been disseminated through schools as well as pediatric and community initiatives in in high-incidence areas, demonstrating scalability and effectiveness when embedded within existing educational frameworks.

Similarly, Noval et al. [26] developed and implemented a standardized community/school intervention for rural adolescents, combining classroom instruction with practical training. The program produced marked improvements in ATV safety knowledge and self-reported protective behaviors among participants. This model highlights the value of local delivery through trusted messengers, such as educators, healthcare workers, and agricultural extension agents, who can tailor the content to local contexts and norms.

The principles and successes of the international interventions detailed above can be adapted to the Israeli context through culturally and demographically tailored community programs:

- Integration into school curriculum:
- Similar to the STARs model, ATV and off-road vehicle safety education can be incorporated into physical education, civics, or road safety programs in middle schools, particularly in rural and peripheral areas.
- Community-led education through trusted institutions:
- Delivery through municipal youth departments, regional councils, and Arab community centers can ensure high local engagement and cultural resonance.
- Parent-focused awareness campaigns:
- Short, culturally adapted video interventions can be disseminated via social media, community events, or healthcare channels to promote helmet use, supervision, and adherence to age restrictions.
- Targeted resource support:
- Subsidies for helmets and protective gear, and the creation of safe, designated riding areas in low-income or rural communities, would address structural inequities that contribute to higher injury risks.
- Cross-sectoral collaboration:
- Partnership among Ministries of Education, Transportation and Health, together with NGOs and local authorities, could sustain ongoing education, enforcement, and evaluation efforts.

### Policy implications and recommendations

International evidence consistently shows that ATVs pose substantial risks to children, primarily due to mismatch between vehicle design and children’s physical capabilities, inadequate protective measures, and insufficient regulatory oversight. These risks are heightened in contexts where legislation is outdated or weakly enforced. Findings from this study reinforce the urgent need for a coordinated, multi-agency policy response in Israel, particularly following recent regulatory rollbacks that have further weakened existing protections.

While we support the current minimum legal age for ATV operation in Israel, this measure is insufficient on its own. The National Program for Child Safety Promotion, operating under the Ministry of Health, is positioned to coordinate national efforts in partnership with relevant ministries and enforcement agencies. To effectively reduce ATV-related injuries and fatalities among children and adolescents, the following policy actions are strongly recommended:

- Establish a uniform, enforceable minimum legal driving age for ATV operation, aligned with international safety standards. The Ministry of Transport and Road Safety should lead the legislative process, in collaboration with the Ministry of Justice, while enforcement should be carried out by the Israel Police and licensing authorities, with additional personnel allocated for field checks and compliance monitoring.
- Mandate installation of Operator Protective Devices (OPDs) on all ATVs, alongside compulsory stability testing and clear consumer labeling. The Ministry of Transport and Road Safety, together with the Standard Institution of Israel, should define the technical specifications. Market surveillance units within the Ministry of Economy and Industry, supported by the customs authorities, should enforce compliance at points of sale and import.
- Strengthen legislative oversight and enforcement mechanisms for ATV sales and usage, including clear penalties for non-compliant retailers and users. Legislative amendments should be overseen jointly by the The Ministry of Transport and Road Safety and the Ministry of Justice. Enforcement responsibilities should fall to the Israel Police and municipal inspectors in high-use localities, with expanded inspection capacity where needed.
- Integrate physical and ergonomic suitability assessments into any licensing or certification process involving minors. The Ministry of Transport and Road Safety should develop standardized assessment criteria, with implementation by licensed examiners in local licensing offices. Training may be required to ensure examiners can reliably evaluate minors’ capability to safely control ATVs.
- Expand targeted public education and awareness campaigns, with a focus on rural and high-risk areas. The Ministry for Social Equality should lead culturally tailored programming, in collaboration with the Ministry of Education and the National Program for Child Safety Promotion. Activities may include school-based modules, workshops for parents, and Arabic-language media campaigns.
- Mandate child-specific safety technologies on ATVs, such as speed limiters, rollover alerts, and remote disabling features. Technical requirements should be defined by the Ministry of Transport and Road Safety and the Standards Institution of Israel. Compliance should be monitored by market supervision units within the Ministry of Economy and Industry, in coordination with importers, manufacturers, and retailers.

In addition to regulatory and enforcement measures, we recommend incorporating ATV safety education into the national school curriculum. The Ministry of Education should lead curriculum development in partnership with the Ministry of Transport and Road Safety and the National Program for Child Safety Promotion. Implementation should focus on high-risk regions, with training provided to educators to ensure consistent and effective delivery.

Because minors’ ATV use is strongly shaped by parental supervision and household norms, Parent-focused messaging should emphasize appropriate vehicle use, supervision, and legal responsibilities. Enforcement mechanisms should include provisions for parental accountability, such as penalties for permitting underage or unlicensed operation.

The marked ethnic and temporal disparities identified in this study underscore the need for culturally tailored prevention strategies. Interventions must reflect differences in community norms, vehicle-use patterns, and enforcement challenges, particularly in rural Arab localities where informal ATV use is common. Collaboration with local leaders, schools, and healthcare providers is essential to tailor safety messages, to build trust, and strengthen compliance.

In contrast to proactive approaches in countries such as Australia and Sweden, Israel’s recent Scaling back of existing regulations represents a concerning policy reversal. Without decisive action by the ministries and agencies outlined above, children, particularly those in vulnerable communities, will continue to face preventable and unacceptable levels of risk from ATV use.

### Limitations

This study is based on data from Beterem Safe Kids Israel’s media-based pediatric injury surveillance system, which, while systematically maintained and validated, does not capture all ATV-related injuries. Minor and moderate injuries are less likely to be reported in the media, and even some severe or, to a lesser extent, fatal cases, may be underrepresented. As such, the actual burden of ATV-related child injuries in Israel is likely to be considerably higher than reported. Morover, the degree of under-reporting may vary by injury severity, geographic region, or population group, potentially introducing bias in the observed distributions. Therefore, the findings and conclusions should be interpreted with caution. Nonetheless, this limitation strengthen, rather than weakens, the core message of this study: that the true scale of ATV-related harm among children and adolescents is likely greater than documented, underscoring the urgency for immediate and comprehensive preventive action.

## Conclusion

The persistent rise in ATV-related injuries and fatalities among children in Israel reflects a preventable and deeply concerning public health failure. Our findings show that current regulations, enforcement practices, and public awareness efforts are insufficient, particularly in rural and socioeconomically disadvantaged communities. Implementing a uniform minimum age for operation, mandating protective technologies, strengthening enforcement, and investing in culturally tailored education are essential steps. Without immediate and coordinated action, the upward trajectory of harm will continue, placing more children at unnecessary and unacceptable risk.

## Data Availability

Data will be provided upon request.
